# Supplementary material for: Pyruvate Dehydrogenase Kinase 1 inhibition mediated oxidative phosphorylation enhancement in cartilage promotes osteoarthritis progression
Source: BMC Musculoskelet Disord. 2023 Jul 20;24:597. doi: 10.1186/s12891-023-06585-6 (PMC10357736; doi:10.1186/s12891-023-06585-6)
Supplement: Supplementary file 1 — Additional file 1. [file 12891_2023_6585_MOESM1_ESM.zip › KEGG.Gsea.1653622432464/KEGG_GLYCEROPHOSPHOLIPID_METABOLISM.html]

Details for gene set KEGG\_GLYCEROPHOSPHOLIPID\_METABOLISM[GSEA]

|  || Dataset | 55457\_collapsed\_to\_symbols.cls.cls #OA\_versus\_normal.cls.cls #OA\_versus\_normal\_repos |
| Phenotype | cls.cls#OA\_versus\_normal\_repos |
| Upregulated in class | normal |
| GeneSet | KEGG\_GLYCEROPHOSPHOLIPID\_METABOLISM |
| Enrichment Score (ES) | -0.33048114 |
| Normalized Enrichment Score (NES) | -1.3318768 |
| Nominal p-value | 0.070491806 |
| FDR q-value | 0.27780303 |
| FWER p-Value | 0.999 |
Table: GSEA Results Summary

  

Fig 1: Enrichment plot: KEGG\_GLYCEROPHOSPHOLIPID\_METABOLISM      
 Profile of the Running ES Score & Positions of GeneSet Members on the Rank Ordered List

  

| SYMBOL | TITLE | RANK IN GENE LIST | RANK METRIC SCORE | RUNNING ES | CORE ENRICHMENT || 1 | PLA2G12A | phospholipase A2 group XIIA [Source:HGNC Symbol;Acc:HGNC:18554] | 600 | 0.623 | -0.0156 | No |
| 2 | AGPAT1 | 1-acylglycerol-3-phosphate O-acyltransferase 1 [Source:HGNC Symbol;Acc:HGNC:324] | 907 | 0.540 | -0.0123 | No |
| 3 | GNPAT | glyceronephosphate O-acyltransferase [Source:HGNC Symbol;Acc:HGNC:4416] | 1026 | 0.512 | 0.0041 | No |
| 4 | PLPP1 | phospholipid phosphatase 1 [Source:HGNC Symbol;Acc:HGNC:9228] | 1109 | 0.494 | 0.0224 | No |
| 5 | CDIPT | CDP-diacylglycerol--inositol 3-phosphatidyltransferase [Source:HGNC Symbol;Acc:HGNC:1769] | 1586 | 0.416 | 0.0062 | No |
| 6 | PLA2G15 | phospholipase A2 group XV [Source:HGNC Symbol;Acc:HGNC:17163] | 1811 | 0.382 | 0.0079 | No |
| 7 | ETNK1 | ethanolamine kinase 1 [Source:HGNC Symbol;Acc:HGNC:24649] | 2196 | 0.339 | -0.0051 | No |
| 8 | DGKQ | diacylglycerol kinase theta [Source:HGNC Symbol;Acc:HGNC:2856] | 2516 | 0.302 | -0.0148 | No |
| 9 | ACHE | acetylcholinesterase (Cartwright blood group) [Source:HGNC Symbol;Acc:HGNC:108] | 2642 | 0.286 | -0.0103 | No |
| 10 | PLA2G4A | phospholipase A2 group IVA [Source:HGNC Symbol;Acc:HGNC:9035] | 2656 | 0.284 | 0.0030 | No |
| 11 | DGKI | diacylglycerol kinase iota [Source:HGNC Symbol;Acc:HGNC:2855] | 2774 | 0.273 | 0.0075 | No |
| 12 | PLA2G10 | phospholipase A2 group X [Source:HGNC Symbol;Acc:HGNC:9029] | 3315 | 0.220 | -0.0236 | No |
| 13 | CDS1 | CDP-diacylglycerol synthase 1 [Source:HGNC Symbol;Acc:HGNC:1800] | 3359 | 0.216 | -0.0161 | No |
| 14 | CDS2 | CDP-diacylglycerol synthase 2 [Source:HGNC Symbol;Acc:HGNC:1801] | 3361 | 0.215 | -0.0054 | No |
| 15 | PLA2G3 | phospholipase A2 group III [Source:HGNC Symbol;Acc:HGNC:17934] | 3648 | 0.190 | -0.0181 | No |
| 16 | CHPT1 | choline phosphotransferase 1 [Source:HGNC Symbol;Acc:HGNC:17852] | 3911 | 0.163 | -0.0304 | No |
| 17 | PEMT | phosphatidylethanolamine N-methyltransferase [Source:HGNC Symbol;Acc:HGNC:8830] | 4496 | 0.113 | -0.0702 | No |
| 18 | LPCAT4 | lysophosphatidylcholine acyltransferase 4 [Source:HGNC Symbol;Acc:HGNC:30059] | 4615 | 0.104 | -0.0742 | No |
| 19 | ADPRM | "ADP-ribose/CDP-alcohol diphosphatase, manganese dependent [Source:HGNC Symbol;Acc:HGNC:30925]" | 4653 | 0.100 | -0.0721 | No |
| 20 | PTDSS1 | phosphatidylserine synthase 1 [Source:HGNC Symbol;Acc:HGNC:9587] | 4784 | 0.088 | -0.0778 | No |
| 21 | PLA2G2D | phospholipase A2 group IID [Source:HGNC Symbol;Acc:HGNC:9033] | 5056 | 0.069 | -0.0955 | No |
| 22 | GPD1L | glycerol-3-phosphate dehydrogenase 1 like [Source:HGNC Symbol;Acc:HGNC:28956] | 5381 | 0.041 | -0.1187 | No |
| 23 | AGPAT2 | 1-acylglycerol-3-phosphate O-acyltransferase 2 [Source:HGNC Symbol;Acc:HGNC:325] | 5545 | 0.029 | -0.1299 | No |
| 24 | ETNK2 | ethanolamine kinase 2 [Source:HGNC Symbol;Acc:HGNC:25575] | 5897 | 0.002 | -0.1571 | No |
| 25 | LPCAT1 | lysophosphatidylcholine acyltransferase 1 [Source:HGNC Symbol;Acc:HGNC:25718] | 5957 | -0.002 | -0.1617 | No |
| 26 | MBOAT7 | membrane bound O-acyltransferase domain containing 7 [Source:HGNC Symbol;Acc:HGNC:15505] | 6134 | -0.015 | -0.1746 | No |
| 27 | LPGAT1 | lysophosphatidylglycerol acyltransferase 1 [Source:HGNC Symbol;Acc:HGNC:28985] | 6519 | -0.043 | -0.2023 | No |
| 28 | PCYT2 | "phosphate cytidylyltransferase 2, ethanolamine [Source:HGNC Symbol;Acc:HGNC:8756]" | 6637 | -0.052 | -0.2088 | No |
| 29 | PLA2G5 | phospholipase A2 group V [Source:HGNC Symbol;Acc:HGNC:9038] | 6772 | -0.061 | -0.2162 | No |
| 30 | DGKB | diacylglycerol kinase beta [Source:HGNC Symbol;Acc:HGNC:2850] | 6801 | -0.063 | -0.2152 | No |
| 31 | DGKG | diacylglycerol kinase gamma [Source:HGNC Symbol;Acc:HGNC:2853] | 6938 | -0.075 | -0.2221 | No |
| 32 | GPD2 | glycerol-3-phosphate dehydrogenase 2 [Source:HGNC Symbol;Acc:HGNC:4456] | 7206 | -0.097 | -0.2380 | No |
| 33 | GPD1 | glycerol-3-phosphate dehydrogenase 1 [Source:HGNC Symbol;Acc:HGNC:4455] | 7420 | -0.112 | -0.2490 | No |
| 34 | PLA2G2E | phospholipase A2 group IIE [Source:HGNC Symbol;Acc:HGNC:13414] | 7486 | -0.117 | -0.2482 | No |
| 35 | DGKA | diacylglycerol kinase alpha [Source:HGNC Symbol;Acc:HGNC:2849] | 7570 | -0.125 | -0.2484 | No |
| 36 | DGKE | diacylglycerol kinase epsilon [Source:HGNC Symbol;Acc:HGNC:2852] | 8208 | -0.172 | -0.2895 | No |
| 37 | PLA2G1B | phospholipase A2 group IB [Source:HGNC Symbol;Acc:HGNC:9030] | 8232 | -0.173 | -0.2826 | No |
| 38 | CHKB | choline kinase beta [Source:HGNC Symbol;Acc:HGNC:1938] | 8413 | -0.186 | -0.2873 | No |
| 39 | AGPAT4 | 1-acylglycerol-3-phosphate O-acyltransferase 4 [Source:HGNC Symbol;Acc:HGNC:20885] | 8577 | -0.200 | -0.2900 | No |
| 40 | CHAT | choline O-acetyltransferase [Source:HGNC Symbol;Acc:HGNC:1912] | 8591 | -0.200 | -0.2809 | No |
| 41 | LYPLA1 | lysophospholipase 1 [Source:HGNC Symbol;Acc:HGNC:6737] | 9023 | -0.234 | -0.3028 | No |
| 42 | PLD1 | phospholipase D1 [Source:HGNC Symbol;Acc:HGNC:9067] | 9365 | -0.261 | -0.3163 | Yes |
| 43 | LPCAT3 | lysophosphatidylcholine acyltransferase 3 [Source:HGNC Symbol;Acc:HGNC:30244] | 9461 | -0.270 | -0.3102 | Yes |
| 44 | PLD2 | phospholipase D2 [Source:HGNC Symbol;Acc:HGNC:9068] | 9615 | -0.284 | -0.3079 | Yes |
| 45 | PLPP3 | phospholipid phosphatase 3 [Source:HGNC Symbol;Acc:HGNC:9229] | 9906 | -0.312 | -0.3148 | Yes |
| 46 | MBOAT2 | membrane bound O-acyltransferase domain containing 2 [Source:HGNC Symbol;Acc:HGNC:25193] | 10038 | -0.325 | -0.3088 | Yes |
| 47 | PLA2G2A | phospholipase A2 group IIA [Source:HGNC Symbol;Acc:HGNC:9031] | 10152 | -0.336 | -0.3007 | Yes |
| 48 | TAFAZZIN | "tafazzin, phospholipid-lysophospholipid transacylase [Source:HGNC Symbol;Acc:HGNC:11577]" | 10429 | -0.364 | -0.3040 | Yes |
| 49 | DGKZ | diacylglycerol kinase zeta [Source:HGNC Symbol;Acc:HGNC:2857] | 10530 | -0.376 | -0.2929 | Yes |
| 50 | PLA2G2F | phospholipase A2 group IIF [Source:HGNC Symbol;Acc:HGNC:30040] | 10628 | -0.386 | -0.2812 | Yes |
| 51 | LYPLA2 | lysophospholipase 2 [Source:HGNC Symbol;Acc:HGNC:6738] | 10860 | -0.411 | -0.2786 | Yes |
| 52 | PLA2G6 | phospholipase A2 group VI [Source:HGNC Symbol;Acc:HGNC:9039] | 11128 | -0.446 | -0.2770 | Yes |
| 53 | PLPP2 | phospholipid phosphatase 2 [Source:HGNC Symbol;Acc:HGNC:9230] | 11160 | -0.452 | -0.2568 | Yes |
| 54 | LCAT | lecithin-cholesterol acyltransferase [Source:HGNC Symbol;Acc:HGNC:6522] | 11514 | -0.503 | -0.2592 | Yes |
| 55 | PCYT1A | "phosphate cytidylyltransferase 1A, choline [Source:HGNC Symbol;Acc:HGNC:8754]" | 11900 | -0.572 | -0.2605 | Yes |
| 56 | AGPAT3 | 1-acylglycerol-3-phosphate O-acyltransferase 3 [Source:HGNC Symbol;Acc:HGNC:326] | 12062 | -0.611 | -0.2425 | Yes |
| 57 | JMJD7-PLA2G4B | JMJD7-PLA2G4B readthrough [Source:HGNC Symbol;Acc:HGNC:34449] | 12076 | -0.614 | -0.2128 | Yes |
| 58 | DGKD | diacylglycerol kinase delta [Source:HGNC Symbol;Acc:HGNC:2851] | 12222 | -0.651 | -0.1915 | Yes |
| 59 | PGS1 | phosphatidylglycerophosphate synthase 1 [Source:HGNC Symbol;Acc:HGNC:30029] | 12241 | -0.656 | -0.1600 | Yes |
| 60 | PTDSS2 | phosphatidylserine synthase 2 [Source:HGNC Symbol;Acc:HGNC:15463] | 12431 | -0.717 | -0.1389 | Yes |
| 61 | CHKA | choline kinase alpha [Source:HGNC Symbol;Acc:HGNC:1937] | 12511 | -0.751 | -0.1074 | Yes |
| 62 | PISD | phosphatidylserine decarboxylase [Source:HGNC Symbol;Acc:HGNC:8999] | 12581 | -0.785 | -0.0735 | Yes |
| 63 | DGKH | diacylglycerol kinase eta [Source:HGNC Symbol;Acc:HGNC:2854] | 12619 | -0.809 | -0.0358 | Yes |
| 64 | PCYT1B | "phosphate cytidylyltransferase 1B, choline [Source:HGNC Symbol;Acc:HGNC:8755]" | 12877 | -1.151 | 0.0018 | Yes |
Table: GSEA details [plain text format]

  

Fig 2: KEGG\_GLYCEROPHOSPHOLIPID\_METABOLISM      
 Blue-Pink O' Gram in the Space of the Analyzed GeneSet

  

Fig 3: KEGG\_GLYCEROPHOSPHOLIPID\_METABOLISM: Random ES distribution      
 Gene set null distribution of ES for **KEGG\_GLYCEROPHOSPHOLIPID\_METABOLISM**
